# Hydatid of Morgagni in Young Female: A Rare Case of Acute Abdomen—Case Report and Literature Review

**DOI:** 10.1155/2024/4559795

**Published:** 2024-07-05

**Authors:** Taisia Bollettini, Francesco Molinaro, Alice Benigna, Enrico Ciardini, Gabriele Centini, Natale Calomino, Giulia Fusi, Messina Mario, Rossella Angotti

**Affiliations:** ^1^ Pediatric Surgery University of Siena, Siena, Italy; ^2^ Pediatric Surgery Santa Chiara Hospital, Trento, Italy; ^3^ Obstetric and Gynecology University of Siena, Siena, Italy; ^4^ General Surgery University of Siena, Siena, Italy

## Abstract

Torsion of the hydatid of Morgagni is a rare condition which can cause acute abdominal pain in young female. We present a case report of a 13-year-old girl with acute abdominal pain and treated for torsion of the hydatid of Morgagni. Less than 20 cases of female younger than 18 years old with this condition are been described in the literature. Through our systematic review performed following the PRISMA statement, we want to emphasize the difficulty in diagnosis and the importance of considering this pathology in the differential diagnosis of acute abdomen in females with the aim of obtaining a timely surgical treatment to preserve fertility in these patients.

## 1. Introduction

Torsion of the hydatids of Morgagni was first reported by Kelly in 1906 [1]. Only 19 cases of girls under 18 years old were reported in the literature since then. The clinical presentation is vague, and preoperative suspicion is generally rare.

Blood and first-level radiological exams are often normal. For these reasons, most of them underwent surgery for acute appendicitis. Other times, surgical intervention is delayed leading to more advanced necrosis and gangrene of the fallopian tube, with serious reproductive consequences. We report the first case associated with sports activity, and we performed a literature review with the aim of identifying the best diagnostic and therapeutic management of this condition.

## 2. Case Report

A 13-year-old and postmenarchal girl came to our emergency department for acute abdominal pain associated with nausea and stranguria.

She is a sporty girl, an athlete, and had been training about 48 h earlier.

Her previous medical and surgical history was not significant. Her last menstrual period was normal 2 weeks previously. She never had gynecologic problems.

Blood and urine exams were normal: white blood cells 7170 mm^3^, N 44%, and PCR < 0.5 mg/dL.

Abdominal examination revealed pain and tender on the right side.

The abdominal ultrasound and Doppler studies of adnexal blood flow were normal.

**Figure 1:**
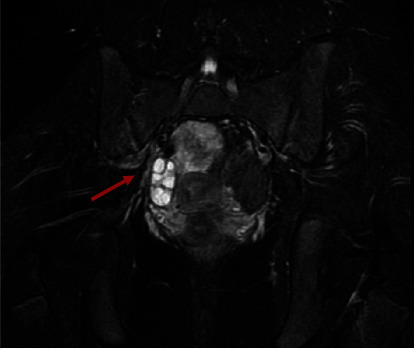
The MRI showed liquid in the Douglas cavity and slight asymmetry of the contrast impregnation in the early phase of the right ovary (arrow) which appeared more visible in the late phase.

An abdomen MRI was done 72 h after the patient's admission in suspicion of adnexal's disease. The MRI showed liquid in the Douglas cavity and slight asymmetry of the contrast impregnation in the early phase of the right ovary which appeared more visible in the late phase (Figure 1).

**Figure 2:**
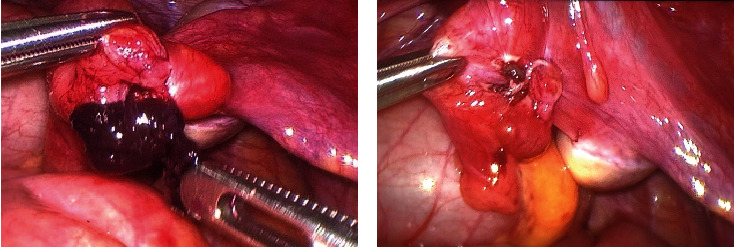
(a) The paratubal necrotic Morgagni hydatid cysts and the distal segment of the fallopian right tube 360° twisted. (b) The right fallopian tube after necrotic Morgagni hydatid cysts resection and derotation.

Urgent laparoscopic exploration was done. We found two small right paratubal Morgagni hydatid cysts, one of them was necrotic, and both were removed (Figure 2).

Moreover, the distal segment of the fallopian right tube was 360° twisted, and we derotated it (Figure 2). The right ovary was not involved in the torsion and appeared normal.

The postoperative course was uneventful, and she was discharged on Day 2.

At 2-month follow-up, she remains well. The patient shared with the team her positive impressions of the diagnostic and treatment management.

Pathological examination confirmed the diagnosis of hydatids of Morgagni.

## 3. Literature Review and Discussion

Female hydatid of Morgagni, described by Morgagni in 1790, is a structure which arise from the fimbriated ends of the fallopian tubes usually smaller in size than paraovarian cysts. It may be multiple or single, and it may be sessile or pedunculated. In the female, both Mullerian and Wolffian remnants can give this kind of cystic structures [2].

Isolated fallopian tube torsion due to twisted Morgagni hydatid is considered to be very rare, with the incidence being 1/1.5 per million cases [3]. However in 2006, Pansky et al. [4] demonstrated that the hydatids of Morgagni were involved in 26% of all cases of adnexal torsion in an adolescent subgroup. They also observed three different possible torsion mechanisms: torsion of the hydatid of Morgagni with intact adnexa, torsion of the adnexa together with torsion of the hydatid of Morgagni, and torsion and entanglement of the hydatid of Morgagni's pedicle around the distal fallopian tube.

In our case, we found a torsion of the hydatid of Morgagni with intact adnexa but with fallopian tube torsion.

In order to achieve the objective of this article, a systematic literature review following the PRISMA criteria was conducted on PubMed and EMBASE using this search strategy: (“hydatid of Morgagni, female”[MeSH] or “isolated tubal torsion”[MeSH] or “Paratubal Cyst Torsion”) and (“Pediatrics”[MeSH] or “Infant”[MeSH] or “Child”[MeSH] or “Adolescent”[MeSH]). The search period was defined from 1947 to the present, and the following inclusion criteria were applied: diagnosis of hydatid of Morgagni torsion, and female patients younger than 18 years old. Cases reported in the literature of isolated torsion of the fallopian tube related to the presence of other types of paratubaric cysts have been excluded [5].

Nine studies followed our eligibility criteria and were included in our review for a total of 19 cases of hydatid of Morgagni torsion in girls younger than 18 years old. We collected data regarding population characteristics, clinical features, diagnosis, and treatment (Table 1).

No cases of girls under the age of 10 have been reported probably because after the onset of puberty, the secretory activity may cause a dilation of the hydatid of Morgagni increasing the risk of torsion.

Reis and De Costa [6] collected 26 cases from 1906 to 1947; we only included six of these patients in our review because the others were more than 18 years old. They describe a common clinical picture: Abdominal or pelvic pain is the most frequent complaint, usually colicky or cramp-like, often diffuse, and later localized to the right or left lower quadrant. No patient appeared very ill. Fifty percent had nausea and vomiting; 25% complained of dysmenorrhea, relieved in the majority by operative removal of the gangrenous cyst.

Also, in our case, the patient presented with acute abdominal pain and was not in serious general conditions. The pain was associated with nausea but also with stranguria, a symptom not described in any case by Reis and De Costa [6].

After this literature review, other 16 cases have been reported: Five of them were premenarcheal girls; the right tube is more frequently involved than the left in torsion; the clinical presentation is similar to that described by Reis and De Costa [6], without specific symptoms.

As already widely described in the literature, isolated tubal torsion is included in the differential diagnosis of acute abdominal pain [5], but torsion of the hydatid of Morgagni in female patients is not. In many of the cases reported in the literature, torsion of the hydatid of Morgagni is associated with torsion of the tuba, which could therefore be the cause of the acute abdominal pain, but in a smaller percentage of cases (26%), a pattern of exclusive torsion of the hydatid of Morgagni associated with acute abdominal pain has been described.

Since 2002, transabdominal ultrasound has been used as a diagnostic aid although it is extremely difficult to identify these cysts because of their small size. Color-Doppler US is helpful to detect the impaired ovarian blood flow however does not exclude the fallopian tube torsion, like in our case.

In the most recent case reported in literature and in our case, an MRI was also performed which provided useful information to decide to proceed with a surgical exploration.

In our case, the MRI was not available in urgency, and since the girl's condition was stable, the examination was scheduled and performed 72 h after the patient's admission.

In any case, the diagnosis of certainty is possible only with surgical exploration and anatomopathological examination of the cyst. This surgical exploration can be performed via either laparotomy or laparoscopy. All cases described from 2006 were treated with a laparoscopic approach guaranteeing a global evaluation of abdomen and good aesthetic results and reducing the postoperative pain and hospitalization.

The treatment described in the literature consists of adnexal-sparing surgery with cystectomy and detorsion of twisted tube if present. Mueller and Tomita [7] treat one of the two cases described with marsupialization of the cyst.

Three salpingectomies are reported in the literature [2, 3, 8] because of necrotic degeneration of fallopian tube: One of them is a partial salpingectomy [3] and another is a salpingo-oophorectomy [2]. Bertozzi et al. [9] reported that conservative management for isolated fallopian tube torsion could also be considered in cases of necrotic tubes because morbidity would not increase, but it could create the possibility of leaving a nonfunctional tube.

Routine appendectomy was also performed in three cases described in the literature [2, 10, 11].

In our case, we have performed laparoscopy cystectomy and detorsion of twisted right tube with tube-sparing surgery. Salpingopexy is not supported by literature even if there is no data for a contraindication. We decided not to perform it.

Adorisio et al. [12] suggest a conservative treatment with anti-inflammatory drugs such as in the torsion of the hydatid of Morgagni in male patients. In our opinion, it could do if the diagnosis is certain. To date, there are no symptoms or diagnostic investigations that allow to exclude the torsion of the fallopian tube before surgical exploration. Therefore, in a patient presenting with acute abdomen and clinical suspicion of Morgagni's hydatid torsion, prompt exploratory laparoscopy is indicated to prevent the progression of necrosis, which could potentially involve the fallopian tube and ovary. Failure to act swiftly may necessitate salpingectomy or salpingo-oophorectomy, compromising the fertility of the young patient. The last necessary consideration is that our patient was very athletic and her symptoms were composed following intense training. This anamnestic data prompted us to perform a second-level investigation in the suspicion of a torsion of the fallopian tube related to workout.

A case of Morgagni's hydatid torsion in female reported in the literature is described in this association. However, it is known that isolated tubal torsion is related to sports and should be considered in patients with abdominal pain who play sports with sudden body movements in absence of clinical suspicion of other cause [13].

## 4. Conclusion

In conclusion, the torsion of the hydatid of Morgagni in young girls must be considered as a possible cause of abdominal or pelvic pain. The relationship between sports and rapid movements of the body, as in athletic girls, is interesting and should be considered.

Color-Doppler study of ovarian blood flow should be always performed in these cases, but this could be normal even when the torsion of the hydatid of Morgagni is associated with torsion of the tube. For this reason, a second-level imaging exam such as abdominal MRI can help for the diagnosis. The main limit of MRI, at least in our reality, is the impossibility to perform it in emergency. In cases where a relatively prompt second-level diagnostic examination is not feasible, laparoscopic exploration is recommended to prevent complications that could reduce the patient's fertility.

Our work can be useful to the paediatric surgeon because it helps to learn more about an uncommon pathology that can cause a very common symptom (abdominal/pelvic pain). Major limitation is represented by the case report nature of the paper; multicenter studies focusing on this condition are needed and could be useful in identifying the most appropriate diagnostic management.

Based on our experience, according to the literature, we recommend an early laparoscopic exploration and an adnexal-sparing treatment when it is possible.

## Figures and Tables

**Table 1:** Literature review: Hydatid of Morgagni torsion in girls younger than 18 years old [2–4, 6–8, 10–12].

**Author**	**N**	**Age (years)**	**Menarca**	**Symptoms**	**Imaging**	**Treatment**	**Tubal torsion**	**Side**
Adorisio et al. [12]	1	Teenager	—	Right lower quadrant abdominal pain, nausea	US: Cystic lesion (27 mm) MRI	VLS, excision	No	Right
Mueller and Tomita [7]	2	11.5	Pre	Pelvic and colicky pain	US	VLS, detorsion, excision-marsupialization of the cyst	Yes	Left/right
Cimador et al. [3]	1	12	Pre	Right lower quadrant abdominal pain	US: Right cystic mass 7 cm	VLS, partial salpingectomy	Yes	BIL (left torsion)
Muthucumaru et al. [11]	2	12.5	Pre	Right lower quadrant abdominal pain; one associated with nausea and vomiting	US: One polycystic ovaries	VLS, torted cyst was excised, appendicectomy	No	Right
Pansky et al. [4]	4	13–18	Post	Localized lower abdominal pain and nausea	US: 3 negative and one with ovarian cysts	VLS detorsion and cystectomy of the hydatid of Morgagni	2 no 2 yes	—
Rizk, Lakshminarasimha, and Joshi [8]	1	17	Post	Right lower quadrant abdominal pain, fever, nausea, and vomiting	US: Right ovarian cyst 3 cm	Laparotomy, right salpingectomy	Yes	Right
Kern [2]	1	13	Post	Intermittent and colicky abdominal pain, nausea, and vomiting	—	Laparotomy left salpingo-oophorectomy and appendicectomy	Yes	Left
Schultz and Chapman [10]	1	18	Post	Right lower quadrant abdominal pain	—	Laparotomy cyst excision, appendicectomy	No	Right
Reis and De Costa 1947 (review from 1906) [6]	6	15.3	—	Right/left lower quadrant abdominal pain cramp-like. Nausea and vomiting (25%)	—	Laparotomy cyst excision		Left/right

## References

[1] Kelly H. A. (1906). *Operative gynecology*.

[2] Kern I. B. (1969). Torsion of the hydatid of Morgagni in the female. *ANZ Journal of Surgery*.

[3] Cimador M., Pensabene M., Siracusa F. (2014). Laparoscopic management of an isolated left fallopian tube torsion due to twisted Morgagni hydatid in a pre-menarcheal girl. *La Pediatria Medica e Chirurgica*.

[4] Pansky M., Smorgick N., Lotan G., Herman A., Schneider D., Halperin R. (2006). Adnexal torsion involving hydatids of Morgagni a rare cause of acute abdominal pain in adolescents. *The American College of Obstetricians and Gynecologists*.

[5] Liang Q., Xue W., Li D., Li S., Ding J. (2021). Isolated fallopian tube torsion with paraovarian cysts: a case report and literature review. *BMC Women’s Health*.

[6] Reis R. A., De Costa E. J. (1948). Torsion of the Hydatid of Morgagni. *American Journal of Obstetrics & Gynecology*.

[7] Mueller C., Tomita S. (2016). Fallopian tube torsion as a cause of acute pelvic pain in adolescent females. *Hindawi Publishing Corporation Case Reports in Pediatrics*.

[8] Rizk D. E., Lakshminarasimha B., Joshi S. (2002). Torsion of the fallopian tube in an adolescent female. *Journal of Pediatric and Adolescent Gynecology*.

[9] Bertozzi M., Magrini E., Riccioni S., Giovenali P., Appignani A. (2017). Isolated fallopian tube torsion with hydrosalpinx: review of a debated management in a pediatric population. *Journal of Pediatric Surgery*.

[10] Schultz M. H., Chapman H. L. (1950). Torsion of the hydatid of Morgagni. *Canadian Medical Association Journal*.

[11] Muthucumaru M., Yahya Z., Ferguson P., Cheng W. (2011). Torsion of hydatids of Morgagni in premenarchal adolescent girls-a case report and review of literature. *Journal of Pediatric Surgery*.

[12] Adorisio O., Diomedi Camassei F., de Peppo F. (2022). Torsion of the hydatid of Morgagni in a teenage girl. *BMJ Case Reports*.

[13] Bertozzi M., Noviello C., Molinaro F. (2020). Isolated fallopian tube torsion in pediatric age: an Italian multicenter retrospective study. *Journal of Pediatric Surgery*.

